# β-Carboline alkaloids induce structural plasticity and inhibition of SARS-CoV-2 nsp3 macrodomain more potently than remdesivir metabolite GS-441524: computational approach

**DOI:** 10.3906/biy-2106-64

**Published:** 2021-08-30

**Authors:** Yusuf Oloruntoyin AYIPO, Sani Najib YAHAYA, Halimah Funmilayo BABAMALE, Iqrar AHMAD, Harun PATEL, Mohd Nizam MORDI

**Affiliations:** 1 Centre for Drug Research, Universiti Sains Malaysia, Pulau Pinang Malaysia; 2 Department of Chemical, Geological and Physical Sciences, Kwara State University, Ilorin Nigeria; 3 Department of Pharmaceutical and Medicinal Chemistry, Bayero University, Kano Nigeria; 4 School of Chemical Sciences, Universiti Sains Malaysia, Pulau Pinang Malaysia; 5 Department of Industrial Chemistry, University of Ilorin, Ilorin Nigeria; 6 Department of Pharmaceutical Chemistry, R. C. Patel Institute of Pharmaceutical Education and Research, Maharashtra India

**Keywords:** SARS-CoV-2, nsp3 macrodomain, ADP-ribose, β-carboline, bioinformatics, bioinformatics, drug design

## Abstract

The nsp3 macrodomain is implicated in the viral replication, pathogenesis and host immune responses through the removal of ADP-ribosylation sites during infections of coronaviruses including the SARS-CoV-2. It has ever been modulated by macromolecules including the ADP-ribose until Ni and co-workers recently reported its inhibition and plasticity enhancement unprecedentedly by **remdesivir** metabolite, **GS-441524**, creating an opportunity for investigating other biodiverse small molecules such as β-Carboline (βC) alkaloids. In this study, 1497 βC analogues from the HiT2LEAD chemical database were screened, using computational approaches of Glide XP docking, molecular dynamics simulation and pk-CSM ADMET predictions. Selectively, βC ligands, **129, 584**, **1303 **and **1323** demonstrated higher binding affinities to the receptor, indicated by XP docking scores of –10.72, –10.01, –9.63 and –9.48 kcal/mol respectively than **remdesivir** and **GS-441524** with –4.68 and –9.41 kcal/mol respectively. Consistently, their binding free energies were –36.07, –23.77, –24.07 and –17.76 kcal/mol respectively, while **remdesivir** and **GS-441524 **showed –21.22 and –24.20 kcal/mol respectively. Interestingly, the selected βC ligands displayed better stability and flexibility for enhancing the plasticity of the receptor than **GS-441524**, especially **129 **and **1303**. Their predicted ADMET parameters favour druggability and low expressions for toxicity. Thus, they are recommended as promising adjuvant/standalone anti-SARS-CoV-2 candidates for further study.**Key words**: SARS-CoV-2, nsp3 macrodomain, ADP-ribose, β-carboline, bioinformatics, drug design

## 1. Introduction

**Figure 1:**
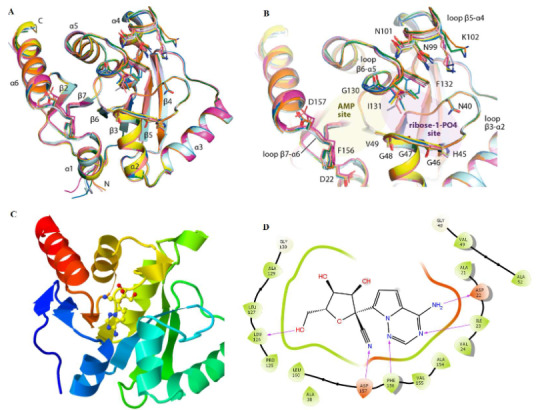
Illustration of the ADP-ribose binding pocket of nsp3 macrodomain of SARS-CoV-2. (A) Plasticity of the ADP-ribose binding pocket; (B) Superimposition of eight molecules from the asymmetric units of two distinct apo crystal structures (PDBs 6YWK and 6YWM) (Reprinted with permission from (Ni et al., 2021), Copyright (2021) American Chemical Society; (C) 3D view of nssp3 Mac-1 in complex with G-441524 (PDB 7BF6); (D) Binding pose showing interactions of G-441524 with amino acids at the active ADP-ribose binding site.

The non-structural protein 3 (nsp3) remains the largest interactive protein complex of coronaviruses (CoVs) with several macrodomains (Macs) located in the open reading frame (ORF) 1ab. The first macrodomain (Mac-1) encodes genome essential for the regulatory activity of the host immune responses through the recognition and removal of posttranslational adenosine diphosphate- (ADP-)ribosylation specific sites during infection (Ni et al., 2021). It is, therefore, implicated in the pathogenesis and as a virulence factor in all forms of human CoV infections (Alhammad et al., 2020). Several positive-stranded genomic RNA viruses including the severe acute respiratory syndrome coronavirus 1 (SARS-CoV-1), Middle East respiratory syndrome coronavirus (MERS-CoV) and SARS-CoV-2 exhibit the encoding of a structurally conserved Mac-1 module that binds and removes ADP-ribose (Lin et al., 2020; Ayipo et al., 2021). The progression of the process induces pathogenic disruption of the host innate immune system through its mono(ADP-ribosyl) hydrolase activity, making an inhibitory binding of bioactive scaffolds to its adenosyl active pockets a prime therapeutic pathway (Cho et al., 2016; Alhammad et al., 2020; Babar et al., 2020; Brosey et al., 2021). These mechanistic posttranslational modifications by nsp3 Mac-1 occurring as poly- or mono-ADP-ribosylation (MARYlation) are catalysed by several enzymes, notably, mono-ADP-ribosyl transferase (MART), and are hypothetically associated with stress expression and innate immune response against viral pathogenesis. The activation of several poly-ADP-ribose polymerases (PARPs) in response to molecular patterns and interferon pretreatments also support MARYlation as an essential modification for the innate immune response against viral infections, including the current pandemic, coronavirus 2019 (COVID-19) (Challa et al., 2021; Ni et al., 2021). Therefore, the associated expressions of the viral life cycle (replication) and infection, as well as the deregulation of host immune responses, make the nsp3 Mac-1 a plausible therapeutic target for effective prophylaxis and treatment of SARS-CoV infections. Moreover, structural and experimental evidence strongly emphasize the contributions of its plasticity to the development of potent inhibitors. Notably, large bioactive molecules, including ADP-ribose, have ever been commonly reported as an ideal candidate for this prospect (Figure 1A). Other potent inhibitors of the nsp3 Mac-1 of SARS-CoV-2 of this type, including HEPES and MES8-4, have also been reported (Brosey et al., 2021). From experimental reports, other active biodiverse small molecules targeting Mac-1 of CoVs are suggested to possess interesting broad anti-CoV activity (Cho et al., 2016; Alhammad et al., 2020). Subsequently, some X-ray-resolved structures of the nsp3 Mac-1 of SARS-CoV-2 have been documented in Protein Data Bank (PDB) in complexes with various naturally occurring nucleosides and nucleotides (Figure 1B). A recent comparative analysis of the complexes indicated degrees of domain plasticity of the ADP-ribose binding site of the target influenced by remdesivir metabolites, GS-441524 and its phosphorylated derivative among the tested small bioactive ligands. The intrinsic plasticity of amino acid residues such as Ile131 and Phe132 in the ribose-phosphate binding site supported by Ala129 and Gly130 main chain flips highly potentiate the availability of the site for the binding of small molecules such as GS-441524 (Figures 1C-D) (Ni et al., 2021). Interestingly, remdesivir and its GS-441524 metabolite were renown active inhibitors of the viral replication through the RNA-dependent RNA polymerase (RdRp). However, the experimental data in the study by Ni et al., unprecedentedly indicates an improved conformational complementarity with the binding pocked in complex with GS-441524 compared to ADP-ribose and suggests an enhanced plasticity of the nsp3 macrodomain influenced by its structural and physicochemical features (Ni et al., 2021). The interesting experimental results obtained by the novel study provides an opportunity for the investigation of other biodiverse small molecules as plasticity enhancers and inhibitors of nsp3 macrodomain of SARS-CoV-2.

β-Carboline (βC) alkaloids are structurally diverse organic small molecules with tricyclic pyrido [3,4-b] indole scaffolds. They exist in vast natural products and as interesting synthetic analogues renowned to medicinal chemists. Their structural features consist of pharmacophoric sites favouring interaction with various biological targets. As such, numerous molecular derivatives of βC analogues have been reported with impressive pharmacology as antimicrobial, anticancer and antiviral agents among others. Specifically, their disruptive intercalating ability on DNA and/or RNA support inhibitory potentials against tumour and viral cells (Ayipo et al., 2021; Ayipo Y et al., 2021; Chakravarti et al., 2021). Subsequently, the inhibitory effects of some natural βC analogues have been documented against some targets implicated in the pathogenesis and treatment of COVID-19 including the viral main proteases, spike glycoprotein, nsp9 and host angiotensin-converting enzyme 2 (ACE2) as well as the transmembrane protease serine 2 (TMPRSS2) (Chakravarti et al., 2021; Parmar et al., 2021). Despite the impressive activity against other CoV therapeutic targets, the evaluation of their potency as promising anti-CoV agents specifically with mechanistic inhibition and plasticity enhancement of nsp3 Mac-1 remains unreported from our findings.

The applications of computational tools such as molecular docking, molecular dynamics and predictors of physicochemical, pharmacokinetics and toxicological profiles of drug-like candidates are not only powerful but have become invaluable in modern drug designs. They have been reported as faster, cost-effective and environmentally friendly methods for identifying lead-like candidates for further experimental study. The applications have been reported with numerous successes leading to the discovery of various FDA-approved medications (Meng et al., 2011; Ruyck and Brysbaert, 2016; Phillips et al., 2018).

**Figure 2:**
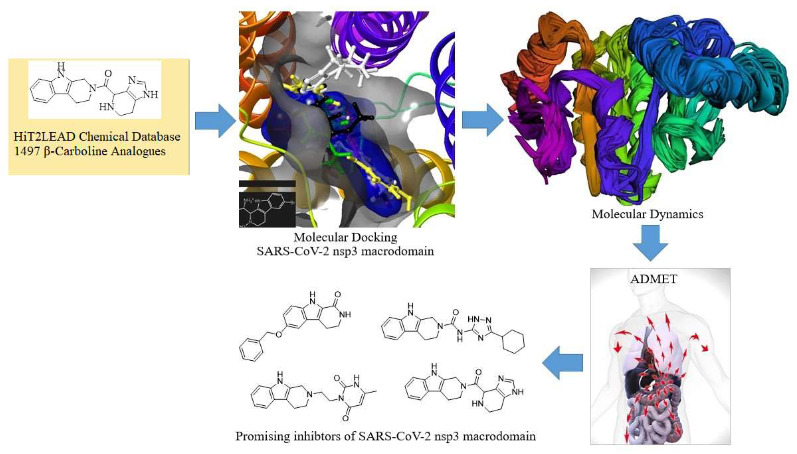
Workflow of the study.

Thus, in this study, βC analogues were retrieved from the HiT2LEAD chemical database and screened for prospective inhibitors and plasticity enhancers of SARS-CoV-2 nsp3 macrodomain, using computational approaches of Maestro Glide XP docking, MD simulation of Ligand and Receptor Molecular Dynamics (LARMD) server-based software. The pharmacokinetics, druggability and safety of the selected potentials were assessed about remdesivir and its metabolite, GS-441524 using pk-CSM ADMET predictions. The workflow is represented in Figure 2.

## 2. Materials and methods

### 2.1. Ligand selection

The HiT2LEAD chemical database was searched for substructures of βC alkaloids. The retrieved βC analogues were downloaded in structure data files (SDFs) for the study.

### 2.2. Ligand preparation

The SDF files of the retrieved βCs and remdesivir and GS-441524 controls were imported into the workspace of Maestro 12.12 (LigPrep, Schrodinger, LCC, New York, NY, USA), followed by preparation through protonation, energy minimization, and estimation of geometry and partial atomic charges using optimized potentials for liquid simulations 3e (OPLS-3e) force field (Harder et al., 2016). The default Epik at a target was set at pH 7.0 ± 2.0 to generate possible ionization states and the minimized conformers, saved in LigPrep.out file.

### 2.3. Protein preparation, binding site analysis and generation of the receptor grid box

The PDB format of the crystal structure of SARS-CoV-2 macrodomain in complex with remdesivir metabolite, GS-441524, expressed in *Escherichia coli *(PDB 7BF6) was retrieved from RCSB PDB. It was preprocessed by assigning bond order, protonation states and filling missing loops using Protein preparation wizard (Madhavi et al., 2013). Further preparation was then followed by optimization, refining and energy minimization for the conversion of heavy atoms to relative mean standard deviation (RMSD) 0.3 using the OPLS-3e force field of Maestro 12.12 (Harder et al., 2016). The enclosing grid box for docking was generated by selecting the centroid of the workspace GS-441524 with x, y and z coordinates of 19.07, –2.54 and 3.83, respectively and default settings of the ligand docking length within 20 Å, saved in gridbox.zip file.

### 2.4. Molecular docking

The ligand-receptor docking simulation was employed to virtually evaluate the inhibitory potentials of the 5191 conformers of βC analogues and reference drugs (positive controls) generated from the LigPrep module against the prepared file of SARS-CoV-2 nsp3 macrodomain (PDB 7BF6). Both the receptor grid zip and ligand output files were uploaded in the Maestro 12.2 Glide docking panel for XP ligand-receptor docking simulation (Halgren et al., 2004). The choice of XP module provides an opportunity for more discriminate, robust torsional refinement and ligand sampling, though it’s more time-consuming and it requires highly configured computing resources (Parmar et al., 2021). The precision, accuracy and reproducibility of the docking protocol were validated through redocking of the co-crystallized ligand within the active pocket of the receptor and computing its RMSD in comparison with the retrieved crystal complex from RCSB PDB. An RMSD value of ≤2.0 Å validates the protocol as good (Castro-Alvarez et al., 2017; Ramírez and Caballero, 2018). The docked ligands were ranked based on the algorithms of docking and glide XP scores, while their interactions with amino acid residues in the active binding pocket of the receptor were analysed using the generated 2D binding poses.

### 2.5. Molecular dynamics

The binding energy, stability and flexibility trajectories of the selected ligand-receptor complexes were evaluated using molecular mechanics (MM) force field in LARMD server-based software integrated with AMBER 16 force field (Yang et al., 2019). The PDB file of each complex system was uploaded for ‘Int-mod’ simulation with default all-atom MD set at 3000 ps, water implicit. The statistics of the H-bonding plot indicates the H-bond contacts that occurred between the ligands and amino acid residues during the simulation. The deviation of the average distance of the atoms of the docked ligands from their original positions was displayed by RMSD plots, while the radius of gyration reveals the RMS average of the distance of all atoms of the receptor/ligand from the centre of mass. The possible conformational dynamics of each molecular complex system and energetics of the binding of ligands were assessed by capturing the transition states of the receptor with a folding free energy barrier, represented by the fractions of native contacts. The fluctuations and isotropic displacements that occurred on each amino acid residue along the simulation trajectories were represented by the RMS fluctuation (RMSF) and B-factor plots. Molecular mechanics generalized born surface area continuum solvation [MM/GB(SA)] re-scoring methods and the statistics of hydrogen bonds between the bound ligands and the protein residues depict the ligand-receptor binding affinities and free energies. The MM/GB(SA) was calculated as ΔG_bind_ in the equations below: 

ΔG*_bind_*= G*_complex_*–G*_receptor_*–G*_ligand_*(i)

 = ΔH - TΔS ≈ ΔE_gas_+ ΔG_sol_+ TΔS (ii)

 ΔE*_gas_*= ΔE*_int_*+ ΔE*_ele_*+ ΔE*_vdw_* (iii)

 ΔG*_sol_*= ΔG*_GB_*+ ΔG*_surf_*(iv)

 where ΔE_gas_ is the gas-phase energy change, ΔE_int_ represents the change in internal energy, ΔE_ele_ is the electrostatic force, ΔE_vdw_ is the change in van der Waals forces, ΔG_sol_ is the solvation free energy, while ΔG_surf_ represents the non-polar contribution to the solvation-accessible surface area (Genheden and Ryde, 2015; Wang et al., 2019; Babar et al., 2020). The βC ligand with the highest stability and ability to induce plasticity when in complex with the receptor target was further subjected to the CABS-flex molecular dynamics module in comparison with GS-441524. The server-based software incorporates a knowledge-based force field and stochastic dynamics of the Monte Carlo method to predict models and trajectories to indicate the flexibility of the protein structure (Jamroz et al., 2013). 

To further study the dynamic evolutions of the ligands and their long time stability in the active binding pocket of the receptor as well as the change in protein structure within a solvent system, 20 ns MD simulation was conducted for ligands 129 and 1303 complexes using the academic version of Desmond 2020-3 (Maestro-Desmond Interoperability Tools, Schrödinger, New York, NY, USA). The solvated system was modelled using the System Builder panel. Each ligand-receptor complex was centred in an orthorhombic cubic box with periodic boundary conditions and filled with Single-point charge (SPC) water molecules buffered at a minimum distance of 10 Å between a protein atom and box edges. The system was neutralized by randomly adding a sufficient number of counter-ions (Na^+^ and Cl^−^), and an isosmotic state was maintained by adding 0.15 M NaCl. It was thereafter minimized and relaxed by OPLS 2005 force field, keeping other parameters of Desmond at default (Ahmad et al., 2021; Jorgensen et al., 1996; Patel et al., 2021). The MD simulations were carried out using an isothermal-isobaric (NPT) ensemble with 300 K temperature, 1 atm pressure, and a 200 ps thermostat relaxation period. A total number of 1000 trajectories were produced within the 20 ns period, and then analysed using the simulation interaction diagram (SID) tool (Kalibaeva et al., 2003; Patel H et al., 2021).

### 2.6. In silico pharmacokinetics, druggability and toxicological assessment

Physicochemical, pharmacokinetics and pharmacodynamics parameters crucial to the delivery of drugs to targets for physiological expressions without significant toxicity were predicted for the selected βCs using the pkCSM-pharmacokinetics server by the input of a string of SMILE files. The module incorporates distance-based graph structural signatures through accurate and precise calculations of atomic pharmacophore frequency counts, toxicophore fingerprints and general molecular properties of novel candidates (Pires et al., 2015). The parameters, including molecular weight (MW), lipophilicity (Log P), solubility (Log S), number of hydrogen bond acceptor/donor (HBA and HBD), number of rotatable bonds (flexibility), Log P, topological polar surface area (TPSA) were quantitatively predicted to profile their absorption, distribution, metabolism, excretion and toxicity (ADMET). The druggability of the selected βCs was also determined using the potentials for blood-brain barrier (BBB), p-glycoprotein (pg) substrate, human gastrointestinal absorption and the inhibitory potentials on cytochrome P450 isoenzymes. The parameters were referenced to the BDDC rules of 5 (RO5) and druggability (Benet et al., 2016).

## 3. Results and discussion

### 3.1 Ligand selection and preparation

The search on Cambridge online chemical database, HiT2LEAD (https://www.hit2lead.com) for βC substructures produced 1497 bioactive analogues, which generated 5191 ready-to-dock protonated conformers.

### 3.2. Molecular docking

**Figure 3:**
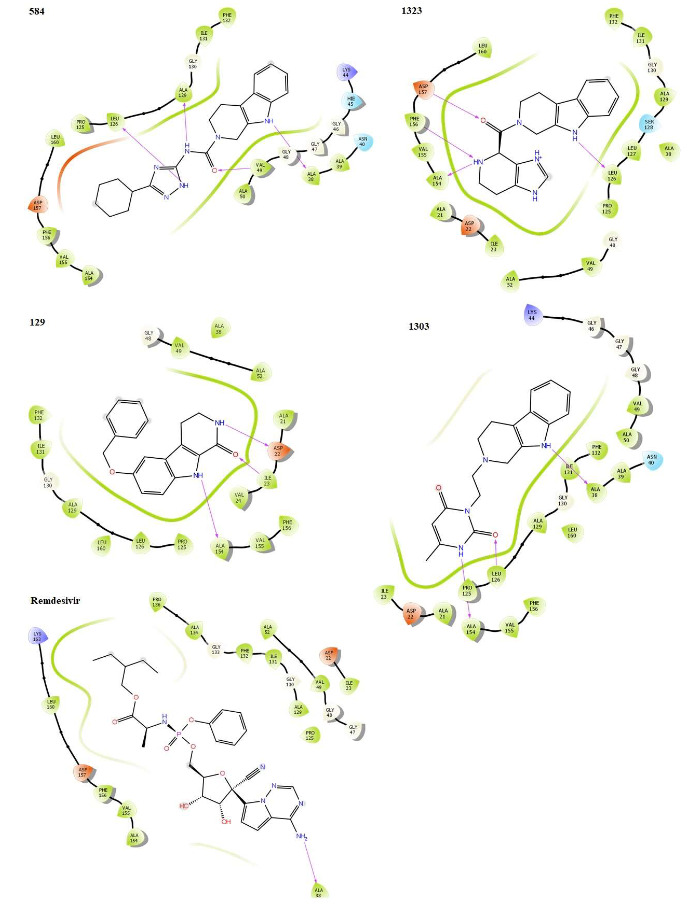
Binding poses showing interactions of selected βC ligands with conserved amino acid residues in the active pocket of SARS-CoV-2 nsp3 macrodomain (PDB 7BF6). Interactions are shown as hydrogen bonding (magenta arrow), π - cation (blue line), salt bridge (red line) and π - π stacking (green line).

The computational evaluation of the potentials of the prepared 5191 βC ligands to inhibit the nsp3 macrodomain of SARS-CoV-2 in references to remdesivir and GS-441524 as described in the experimental section produced the docking results. The redocking of the co-crystallized GS-441524 with the active pocket of the crystal structure PDB 7BF6 generated a pose with an RMSD of 0.51 Å, and the ligand was retrieved among the five best docked poses as expected. The binding interaction shown by the docked ligand was similar to the reported ligand interaction in the RCSB PDB for the receptor, thereby validating the docking protocols as reliable (Castro-Alvarez et al., 2017; Ramírez and Caballero, 2018). From the results (Table 1), it could be observed that four βC analogues 584, 1323, 129 and 1303 orderly show higher binding affinity, a potential for stronger binding inhibitory actions than **remdesivir** and its metabolite **GS-441524,** as indicated by docking score and XP GScore. According to Ni et al., the apo state crystallization of the macrodomain produced two different orthorhombic crystals of the receptor with three and five molecules respectively in the asymmetric units, contributed by Leu131 and Phe132. These potentiate the plasticity of the receptor and favour the binding of small molecules. Additionally, Asp22, Ala38, Val49, Leu126, Leu131, Phe132, Val155, Phe156 and Asp157 reportedly constitute the active binding pocket of the receptor for pharmacologically inclined interactions (Babar et al., 2020). From the crystal structure (PDB 7BF6), the binding of **GS-441524** as a small molecule is similar to that of ADP-ribose, where its NH group is sandwiched between Val49 and Val155, forming H-bonding to Asp22. Similar H-bonding interaction was observed in this simulation through OH to Leu126 and weak interactions to backbone Phe156 and Asp157 residues. These confer charge compatibility by its nitrile group with the receptor pocket. From a previous study, **GS-441524** was recorded as most successful among the tested antivirals including **remdesivir** and its contributions to the plasticity of the receptor as well as inhibitory potentials as promising anti-SARS-CoV-2 were confirmed upon biochemical analyses (Ni et al., 2021). The binding pose of the redocked GS-441524 with the same receptor in this study (Figure 1D) shows similar patterns of bonding and non-bonding interactions, strengthening the reliability of the present findings. The binding poses of selected βC ligands 584, 1323, 129 and 1303 against the same receptor are shown in Figure 3. As observed with GS-441524 reference, all the selected βC ligands formed hydrophilic H-bonding interaction with Leu126 through NH or C=O group except 129. The ligand with the highest docking score, 584 also interacted with Val49, Ala38 and Ala129 all through H-bonding to NH or C=O group. Ligand 1323 additionally formed strong H-bonding interaction to the backbone amino acid residues, Phe156 and Asp157, conferring higher compatibility with the receptor pocket compared to **GS-441524** where the interactions were observably weak (Ni et al., 2021). In similarity to ADP-ribose and **GS-441524**, ligand 129 also show H-bonding interaction to the amino acid Asp22, sandwiched between Val49 and Val155. The selected ligands demonstrate similar pharmacological potentials to ADP-ribose and **GS-441524** through binding interactions with the conserved amino acid residues for specificity of the receptor. They demonstrate higher binding affinity than **GS-441524** and impressively pose as small molecule inhibitors of nsp3 macrodomain for further analysis. Since the protein-ligand binding interactions are crucial for drug actions (Klebe, 2013), the selected compounds as well as the references were subjected to MD simulations for further study of molecular interactions with the receptor.

**Table 1:** Binding affinity of selected βC analogues to SARS-CoV-2 nsp3 macrodomain (PDB 7BF6).

Title	XP GScore	EE	VW	GE	GBS	GBT	TS	MMGB(SA) kcal/mol
584	–10.72	–2.60	–55.58	–58.35	12.91	–45.44	9.37	-36.07
1323	–10.01	11.67	–27.68	–6.00	–13.58	–19.59	5.82	–23.77
129	–9.63	–2.75	–34.45	–37.20	6.60	–30.60	6.53	–24.07
1303	–9.48	37.35	–46.29	–8.95	–14.77	–23.71	5.95	–17.76
GS-44	–9.41	–5.78	–39.88	–45.65	12.07	–33.59	9.59	–24.00
Remde.	–4.68	–9.30	–49.38	–58.77	17.68	–31.08	9.86	–21.22

See supplementary file 1 for complete docking results. GS-44 = GS-441524; Remde = Remdesivir; EE = Electrostatic energy; VW = van der Waals contribution; GE = total gas-phase energy; GBS = GB contribution to solvation; GBT = GB total; MMGB(SA) = final binding free energy.

### 3.3. Molecular dynamics

**Figure 4:**
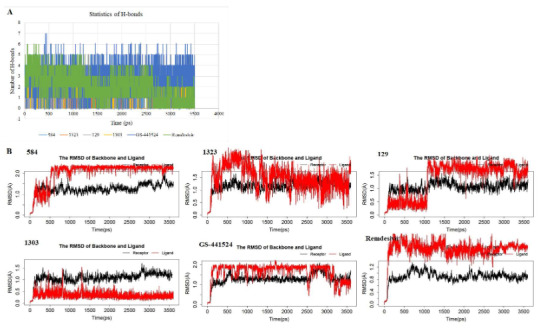
Molecular dynamic simulation trajectories: (A) Statistics of H-bond contacts between the ligands and amino acid residues in the receptor; (B) RMSD of the receptor backbones and the ligands.

The flexibility and thermodynamic stability of the selected ligands in complex with the receptor were virtually studied using MD simulations, an indispensable tool for biophysical and structural probes (Lynch et al., 2017). As observed in Table 1, the total binding free energy, MMGB(SA) deduced by a sum of GBT and TS comprehensively quantify the interactions existing between the selected βC ligands and the SARS-CoV-2 nsp3 macrodomain through various conformations. The results of binding free energies of the selected βC ligands are mostly higher (negative values) than **remdesivir **and** GS-441524**, consistently with the docking scores except for 1303. The statistics of H-bond (Figure 4A) indicates that the **remdesivir** and its metabolite, ** GS-441524** exhibited a higher number of H-bond contacts than the selected βC ligands during the simulations. This should have accounted for higher binding affinities to the receptor especially for **GS-441524** as reported (Ni et al., 2021) than the βC ligands; however, the affinity-inclined contributions of other complementary hydrophilic/hydrophobic bonding and nonbonding interactions to the polar and non-polar amino acid residues, respectively, cannot be overruled. The emphasis on these interactions is essential for their promising druggability (Klebe, 2013). The RMSD plots (Figure 4B) show that the receptor backbone initially experienced a slight atomic deviation of 0.5–1.0 Å and became equilibrated around 100 ps and subsequently with an insignificant deviation of <1.0Å until the end of the simulation in all cases. This supports that the 3500 ps selected for the simulation is adequate for the tentative assignment. The selected βC ligands mostly entered the equilibrium condition around the same time and maintained stability throughout the simulations with insignificant deviations except 1323. Averagely, good thermodynamic stabilities were observed for 584, 129 consistently with the reference **GS-441524**. 

**Figure 5:**
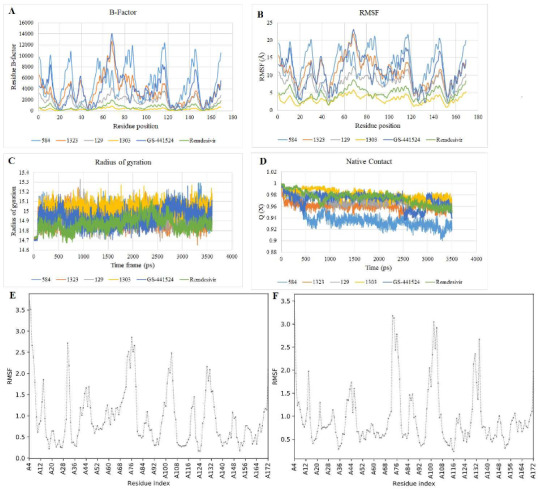
Molecular dynamics simulation results for the selected ligands in complexes SARS-CoV-2 nsp3 macrodomain. (A) The B-/ temperature factor of the selected βC ligands, remdesivir and the GS441524. The lowest fluctuations (Å) are observed with 1303 and 129, indicating the highest stability; (B) RMSF plot of the selected βC ligands and the references. Lower fluctuations are also demonstrated by βC ligands similarly to B-factor, inferring higher stability; (C) Plots of the radius of gyration ligand-receptor complex system. Lower fluctuations indicate more compactness and stability of the ligand-receptor system, relatively favouring βC ligands; (D) Native of contact and (Q) of the ligand-receptor complex. The larger the variations in Q, the more the rigidity of the receptor which is unfavourable for biological functions, especially involving binding of small molecule drugs. The selected βC ligands were also favoured relatively to remdesivir and GS-441524. (E) CABS-Flex RMSF of 129; (F) CABS-Flex RMSF of 129, indicating a reduced RMSF when the receptor was in complex with 129 compared to the complex involving GS-441524.

 The dynamic thermal motion paths, mean square isotropic displacements and transient channels, which support the ligands to enter the internal binding cavities of the receptor are represented by B-factor or temperature factor (Figure 5A) and RMSF plots (Figure 5B). The amino acids with residue numbers 14, 33, 90, 120 and 146 showed the highest fluctuation (between 19 – 21 Å). Although all the selected βC ligands including the references experienced very insignificant fluctuations in complexity with the receptor; however, 129 and 1303 displayed the highest stability indicated by diminutive fluctuation similarly to **remdesivir** but in better forms than **GS-441524**. Since plasticity significantly influences thermodynamic stability, resistance to change in environment and the binding/interaction potentials of protein receptors to ligands, it could be inferred that the enhanced thermodynamic stability observably influenced by the βC ligands support their propensity to enhance the plasticity of the SARS-CoV-2 nsp3 macrodomain as small molecules. These interestingly contribute to the integrity and the predisposition of the target in the pathogenesis of SARS-CoV-2 infection (Panja et al., 2020). To further probe the structure-related influences of the ligands, the compactness and stability of the selected ligand-receptor complexes were observed through the radius of gyration (Rg) plot (Figure 5C). The Rg is determined by the folding state of protein receptors in complexes with the ligands along the trajectories of simulations. All the selected βC ligands, as well as drug references, are favoured by good compactness and stability as indicated by the plot; however, 584 slightly seem perturbed towards the end of the simulation. The native contacts, which capture the transition states between the selected ligands, as well as the reference drugs and the receptor with a folding free energy barrier, indicate the thermostability of the complexes. A system comprising of an unfolding protein is indicated by a large fluctuation in Q (X). Considering the result (Figure 5D), the βC ligands, 1303, 129 and 1323 displayed the least change in Q in the order compared to **remdesivir ** and **GS-441524,** although they all show good stability overall. Thus, selected βC ligands mostly show better contact with the receptor in folding state along the simulations, indicating potentials to influence the flexibility and plasticity of the receptor for binding to small molecule drugs. Since the stability of a protein is dependent on its structural plasticity (Panja et al., 2020), cumulatively, selected βC ligands, especially 1303 and 129 show ideal flexibility, good stability and abilities to enhance the plasticity of the SARS-CoV-2 nsp3 macrodomain for binding to drug molecules in stronger terms than **GS-441524**. Further analyses of the ligand-induced stability and plasticity of the receptor target carried out to compare the impacts of 129 and **GS-441524 **using CABS-flex server showed a reduced RMSF when the receptor was in complex with 129 (Figure 5E) compared to the complex involving **GS-441524 ** (Figure 5F). This also supports the stronger influence of the βC ligands on the stability and plasticity of the receptor compared to **GS-441524**(Jamroz et al., 2013; Tahir ul Qamar et al., 2021). 

**Figure 6:**
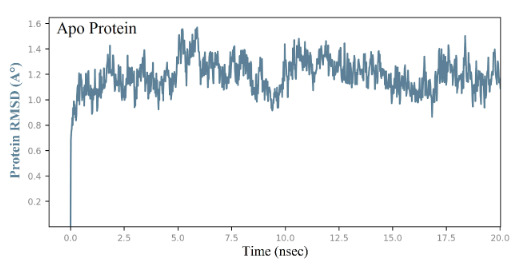
Time-dependent Apo protein RMSD (Å).

**Figure 7:**
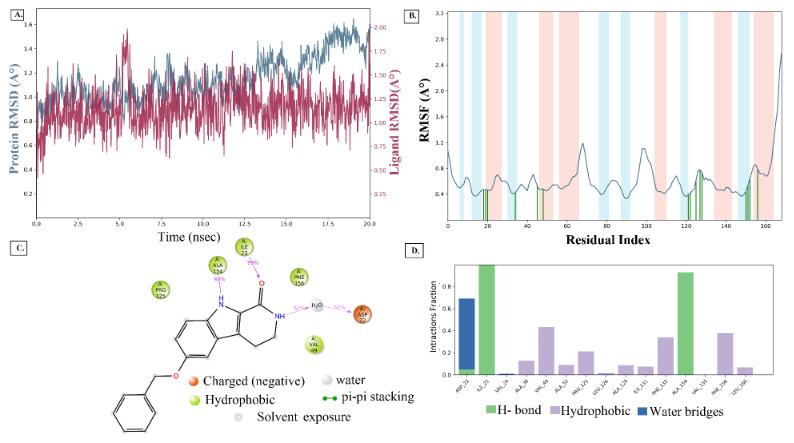
(A) RMSD (Protein RMSD is shown in grey while RMSD of 129 are shown in red) (B) RMSF and (C) 2d contact summary (D) Protein–ligand contact analysis of MD trajectory for 129 complex system.

**Figure 8:**
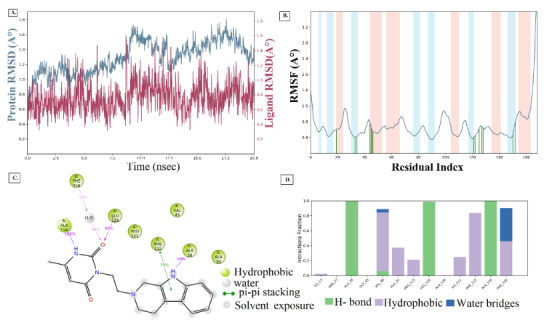
(A) RMSD (Protein RMSD is shown in grey while RMSD of 1303 are shown in red). (B) RMSF and (C) 2d contact summary, (D) Protein–ligand contact analysis of MD trajectory for 1303 complex system.

Upon the extension of MD simulation trajectory to 20 ns for the complexes of ligands 129 and 1303, slight fluctuations within RMSD of 1-3 Å were observed throughout, indicating stable systems (Ahmad et al., 2021; Patel et al., 2021). The Apo protein simulation shows a RMSD range of 1.0 to 1.5 Å (Figure 6), suggesting a good protein stability. The RMSD plot of ligand 129 in complex with the receptor (Figure 7A) indicates a fluctuation of 2.0 Å from the starting position till around 6 ns of the trajectory, which is the highest for the ligand and 1.6 Å for the protein, supporting a stable ligand-protein complex. The RMSF value of the protein backbone residues occurred within 1.2 to 1.6 Å in the catalytic domain, showing a slightly high fluctuation in the C- and N- terminal in contrast to regions of the protein (Figure 7B). During the simulation, the oxygen of ligand 129 accepts hydrogen from Ile23 for 99% of the simulation, whereas the nitrogen of the imidazole group donates hydrogen to Ala154 for 92% of the simulation (Figure 7C). Amino acid residues Asp22, Ile23, and Ala154 exhibited significant hydrogen bonding to ligand 129, played essential roles at the binding pocket, and were considered vital for stabilizing the hit molecules. The amino acid residues, Ala38, Val49, Ala52, Pro125, Leu126, Ala129, Ile131, Phe132, Phe156, and Leu160 showed hydrophobic interaction to 129 (Figure 7D). Similar behaviour was observed with ligand 1303 with RMSD of the Cα backbone (blue) observed between 0.6 to 1.6 Å, and RMSF of 1.2 to 1.6 Å (Figures 8A and 8B). The pyridine-dione carbonyl groups of 1303 primarily interacts with Leu126 through hydrogen bonding and Phe156 via water mediated hydrogen bonding for 98% and 44% of the simulation, respectively (Figure 8C). The histogram depicting the nature of interactions that the protein makes with ligand 1303 throughout the simulation reveals strong hydrogen bonding with Ala38, Val49, Leu126, and Ala154 residues (Figure 8D). The complexes of the SARS-CoV-2 macrodomain and ligand 129 and 1303 were considered to be stable, and the ligands remained within the receptor active pocket throughout the 20 ns simulation.

### 3.4. In silico pharmacokinetics, druggability and toxicological assessment

Physicochemical, pharmacokinetics and pharmacodynamics parameters are crucial to the delivery of drugs to targets for physiological expressions without significant toxicity. The adopted pk-CSM in silico module was validated with several reported experimental results with good accuracy and precision for predicting novel chemical molecules comparable to the time-consuming and expensive experimental modules (Pires et al., 2015). The physicochemical and ADMET properties of the selected βC ligands, **GS-441524** and **remdesivir** are presented in Table 2. The selected ligands have MW in the range of 150–500 g/mol, Log P within the range between –0.7 and +5.0, rotatable bonds between 1–3, <10 HBA, <5 HBD and TPSA 20–130 Å^2^. These physicochemical parameters indicate that the selected ligands have ideal flexibility, lipophilicity, size, solubility, saturation and polarity for good pharmacokinetics of lead-like drug candidates (Pires et al., 2015; Daina et al., 2017). The selected ligands satisfy the BDDC rule of 5 and druggability (Benet et al., 2016). The enhanced flexibility of the selected βC ligands traceable to their number of rotatable bonds could induce more conformational plasticity on the SARS-CoV-2 nsp3 macrodomain than GS-441524 upon binding (Anderson et al., 2001). The ligands and the references are all predicted with good solubility and low permeability across the human intestinal epithelial Caco-2 cell except 129. The selected βC ligands displayed higher intestinal absorption than **remdesivir** and **GS-441524**. They mostly demonstrated no substrate expression for P-glycoprotein, a biological barrier by xenobiotics and extruding toxins, consistently with the references. Their low permeability across the BBB and the central nervous system (CNS) indicate their unideal potentials for CNS drugs, which are unimportant in this particular prospect (NaAllah et al., 2021). Only 1323 showed no expression for metabolism-inclined cytochrome P450 isoenzymes (CYPs) 2D6 and 3A4, similarly to **GS-441524**. They generally signalled good propensity for excretion and low expressions across several pathways for toxicity including hERG I & II inhibition, AMES, *Tetrahymena Pyriformis*, and minnow toxicity. Overall, the predicted physicochemical, pharmacokinetics and pharmacodynamics properties fall within the standards (Pires et al., 2015; Daina et al., 2017) and support their promising potentials as druggable candidates. Although the selected βC ligands mostly demonstrated unimpressive expression to some CYP enzymes along the metabolic pathways, their general toxicity is comparable to the reference drugs.

**Figure 9:**
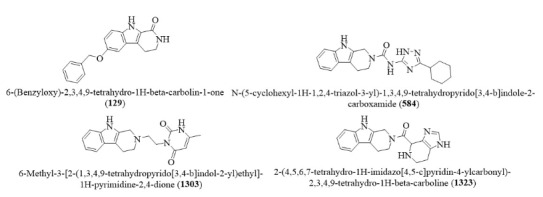
Chemical structures of finally identified βC ligands with potentials for plasticity enhancement and inhibition of nsp3 macrodomain of SARS-CoV-2.

From the screened 1497 βC analogues of Hit2LEAD chemical database, four (4) βC selectively demonstrate promising potentials for enhancing the structural plasticity and inhibiting the nsp3 macrodomain of SARS-CoV-2 in higher terms than remdesivir and GS-441524. They also displayed good physicochemical, pharmacokinetics and pharmacodynamics properties, supporting their druggability with high expression for safety consistently with the reference drugs. More interestingly, the βC alkaloids are vast in natural products across the globe and renowned to scientists with good synthetic accessibility. Experimental validations are required to confirm their bona fide potentials in future studies. However, the selected βC ligands (Figure 9) represent promising candidates for development as adjuvant or standalone therapeutics for effective prophylaxis and treatment of COVID-19 and other emerging strains with mechanistic plasticity enhancement and inhibition of the nsp3 macrodomain target upon further study.

**Table 2:** ADMET properties of selected βC ligands and references.

Property	Descriptor	129	584	1303	1323	GS441525	Remdesivir
MW	(g/mol)	292.338	364.453	324.384	321.384	291.267	602.585
Log P	-	3.0328	3.9239	1.38462	1.6627	–1.85672	2.31218
#Rot. bonds	-	3	2	3	1	2	13
#HBA	-	2	3	4	3	9	13
#HBD	-	2	3	2	3	4	4
TPSA	(Å)2	127.658	157.105	138.295	138.822	118.370	242.488
Absorption	Solubility (mol/L)	–3.57	–3.256	–3.679	–2.826	–2.482	–3.07
Absorption	Caco2 permeability	1.328	0.69	0.765	0.49	0.622	0.635
Absorption	Intestinal (%)	92.866	93.626	95.661	95.783	54.244	71.109
Absorption	Log Kp	–2.773	–2.736	–2.781	–2.735	–2.738	–2.735
Absorption	P-glycoprotein substrate	Yes	Yes	Yes	Yes	No	Yes
Absorption	P-glycoprotein I inhibitor	No	No	No	No	No	No
Absorption	P-glycoprotein II inhibitor	No	Yes	Yes	No	No	No
Distribution	VDss (human)	–0.078	–0.254	0.683	–0.051	–0.262	0.307
Distribution	Fraction unbound	0.04	0.159	0.425	0.437	0.614	0.005
Distribution	BBB permeability	0.134	–1.353	–0.852	–1.077	–1.018	–2.056
Distribution	CNS permeability	–2.046	–2.129	–2.452	–2.495	–3.699	–4.675
Metabolism	CYP2D6 substrate	Yes	Yes	Yes	No	No	No
Metabolism	CYP3A4 substrate	Yes	Yes	Yes	No	No	Yes
Metabolism	CYP1A2 inhibitor	Yes	Yes	Yes	Yes	No	No
Metabolism	CYP2C19 inhibitor	Yes	Yes	No	No	No	No
Metabolism	CYP2C9 inhibitor	No	Yes	No	No	No	No
Metabolism	CYP2D6 inhibitor	No	No	No	No	No	No
Metabolism	CYP3A4 inhibitor	Yes	No	No	No	No	No
Excretion	log mL/min/kg)	0.353	0.079	0.996	1.342	0.513	0.198
Excretion	Renal OCT2 substrate	No	No	Yes	Yes	No	No
Toxicity	AMES toxicity	Yes	No	No	Yes	No	No
Toxicity	Max. tolerated dose (log mg/kg/day)	0.172	0.468	-0.336	0.494	0.718	0.15
Toxicity	hERG I inhibitor	No	No	No	No	No	No
Toxicity	hERG II inhibitor	Yes	Yes	Yes	Yes	No	Yes
Toxicity	Oral Rat Acute Toxicity (LD50) (mol/kg)	2.802	2.709	2.434	2.504	2.939	2.043
Toxicity	Oral Rat Chronic Toxicity (LOAEL) (mg/kg/day)	1.718	2.71	1.629	2.598	3.102	1.639
Toxicity	Hepatotoxicity	Yes	Yes	Yes	No	Yes	Yes
Toxicity	Skin Sensitisation	No	No	No	No	No	No
Toxicity	T. Pyriformis toxicity (µg/L)	0.701	0.286	0.433	0.285	0.284	0.285
Toxicity	Minnow toxicity	3.179	3.823	3.99	2.901	3.306	0.291

Hint: MW = molecular weight; HBA = H-bond acceptor; HBD = H-bond donor; TPSA = topological polar surface area

## 4. Conclusion

The nsp3 macrodomain is strongly implicated in viral replication, host immune response and other pathogenesis of CoV infections including the current COVID-19, as such, represents an important therapeutic target. In this study, computational approaches including molecular docking and molecular dynamics were employed to screen 1497 βC alkaloids from the HiT2LEAD chemical database for prospective inhibitors and plasticity enhancers. Taking advantages of the comprehensive analyses reported against the activity of GS-441524; four βC alkaloids, annotated as 129, 584 1303 and 1323 were identified with interesting potentials to induce more conformational plasticity and inhibition on the nsp3 macrodomain target of SARS-CoV-2 than **GS-441524** along the pharmacological pathway. Selectively, ligands 129 and 1303 possess physicochemical properties that support higher level of stable interactions as inhibitors of the receptor than **GS-441524**. Impressively, they demonstrated good pharmacokinetics and druggability with low expression for toxicity compared to **remdesivir** and **GS-441524**. The results obtained are comparable to the reported literature in favour of GS-441524, which has been validated experimentally. This significantly builds confidence for embarking on a further rigorous experimental investigation of the few identified βC prospects. The ligands are, therefore, recommended as promising anti-SARS-CoV-2 small molecules targeting the nsp3 macrodomain for further translational study. However, further biochemical tests of the molecules suggested as drug candidates in this study should be performed. In conclusion, this study models a faster, cost-effective and environmentally friendly approach to identify promising candidates recommended for translational study into anti-SARS-CoV-2 therapeutics.

## Code availability

Schrodinger suite 2019-4, Build 12 licenced to PD-DRUG-C5357.
